# Stab injury to the preauricular region with laceration of the external carotid artery without involvement of the facial nerve: a case report

**DOI:** 10.1186/s13256-017-1361-9

**Published:** 2017-07-29

**Authors:** Diogo Casal, Giovanni Pelliccia, Diogo Pais, Diogo Carrola-Gomes, Maria Angélica-Almeida, José Videira-Castro, João Goyri-O’Neill

**Affiliations:** 1 Plastic and Reconstructive Surgery Department and Burn Unit, Centro Hospitalar de Lisboa Central, Lisbon, Portugal; 2 Anatomy Department, NOVA Medical School, Universidade NOVA de Lisboa, Campo dos Mártires da Pátria, 130, 1169-056 Lisbon, Portugal; 3 General Surgery Department, Centro Hospitalar de Lisboa Central, Lisbon, Portugal

**Keywords:** Carotid artery, Wound, Stab wound, Trauma, Anatomy, Cadaver, Facial nerve, Case report

## Abstract

**Background:**

Open injuries to the face involving the external carotid artery are uncommon. These injuries are normally associated with laceration of the facial nerve because this nerve is more superficial than the external carotid artery. Hence, external carotid artery lesions are usually associated with facial nerve dysfunction. We present an unusual case report in which the patient had an injury to this artery with no facial nerve compromise.

**Case presentation:**

A 25-year-old Portuguese man sustained a stab wound injury to his right preauricular region with a broken glass. Immediate profuse bleeding ensued. Provisory tamponade of the wound was achieved at the place of aggression by two off-duty doctors. He was initially transferred to a district hospital, where a large arterial bleeding was observed and a temporary compressive dressing was applied. Subsequently, the patient was transferred to a tertiary hospital. At admission in the emergency room, he presented a pulsating lesion in the right preauricular region and slight weakness in the territory of the inferior buccal branch of the facial nerve. The physical examination suggested an arterial lesion superficial to the facial nerve. However, in the operating theater, a section of the posterior and lateral flanks of the external carotid artery inside the parotid gland was identified. No lesion of the facial nerve was observed, and the external carotid artery was repaired. To better understand the anatomical rationale of this uncommon clinical case, we dissected the preauricular region of six cadavers previously injected with colored latex solutions in the vascular system. A small triangular space between the two main branches of division of the facial nerve in which the external carotid artery was not covered by the facial nerve was observed bilaterally in all cases.

**Conclusions:**

This clinical case illustrates that, in a preauricular wound, the external carotid artery can be injured without facial nerve damage. However, no similar description was found in the reviewed literature, which suggests that this must be a very rare occurrence. According to the dissection study performed, this is due to the existence of a triangular space between the cervicofacial and temporofacial nerve trunks in which the external carotid artery is not covered by the facial nerve or its branches.

## Background

The first description of a penetrating neck injury dates back to 5000 years ago in *The Edwin Smith Surgical Papyrus* [1]. Ambroise Paré is credited with the first surgical repair of a major cervical bleeding in the neck region by ligating the carotid artery and the internal jugular vein of a wounded French soldier [1–5]. Nowadays, penetrating head and neck injuries are relatively rare in most countries, representing 2–10% of all trauma admissions [2, 3, 5]. Among these injuries, major arterial lesions are also increasingly infrequent in most developed countries [3]. However, although rare, they can immediately jeopardize life, mandating prompt diagnosis and repair of the severed arteries and neighboring anatomical structures [5–7].

In particular, open injuries to the face involving the external carotid artery (ECA) are relatively uncommon [8]. They are normally associated with compromise of the facial nerve (FN), owing to the more superficial position of this last structure [9]. Hence, ECA lesions are usually associated with FN dysfunction [10]. However, even penetrating injuries to the FN are very rare [11]. For example, in a 16-year retrospective study in which authors reported 456 consecutive patients with 557 peripheral nerve injuries, no cases of open section of the FN in the preauricular region were described [11]. Furthermore, Feldt *et al.*, in reviewing 37,523 head and neck war injuries, found only 35 FN injuries [12].

We present an unusual case report in that the patient presented with an injury to the ECA inside the parotid region with no FN compromise. There is a large consensus in the literature that cases such as this one pose a significant clinical challenge because they involve large and important vascular and/or nerve structures in an emergent scenario [7, 13, 14]. Moreover, clinicians in most centers report limited experience with handling similar cases, which, in turn, causes considerable anxiety in medical personnel [7, 15]. Finally, because these cases are rare, scarce information regarding the surgical anatomy of these wounds is available [16]. The aim of the presentation of this clinical case, as well as of an anatomical dissection study performed to explain the clinical findings observed, is to add to the scant literature on emergent lesions to the ECA and the FN.

## Case presentation

A 25-year-old Portuguese man with an unremarkable medical, social, family, and environmental history sustained a stab wound injury to his right preauricular region with a broken glass while he was sitting in a bar. Immediate profuse bleeding ensued. Provisory tamponade of the wound was achieved at the place of aggression by two off-duty doctors, who pressed a piece of clothing against the wound. The patient was initially transferred to a district hospital, where profuse arterial bleeding was observed by the general surgeon on call. A temporary compressive dressing was applied under local anesthesia. Subsequently, the patient was transferred to a tertiary hospital.

**Fig. 1:**
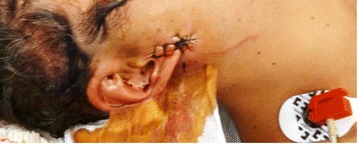
Photograph of the right side of the face of the patient on the operating table, illustrating the right preauricular lesion with a hemostatic suture. Inside the wound, sterile gauze was tamponading the bleeding

At admission in the emergency room, 2 hours after the injury, the patient was slightly diaphoretic and presented with slightly pale skin and mucosae. The patient’s left radial pulse was strong, regular, and had a frequency of 101 beats per minute. The patient presented with a pulsating lesion in the right preauricular region and mild weakness in the territory of the inferior buccal branch of the FN (Fig. 1). The remaining territory of the FN presented no changes. Pin-prick and light touch sensibility in the head and neck did not present changes. There were no signs of dysfunction of the other cranial nerves. The patient’s arterial blood pressure measured in his left arm was 110/75 mmHg. His physical examination was otherwise unremarkable.

**Fig. 2:**
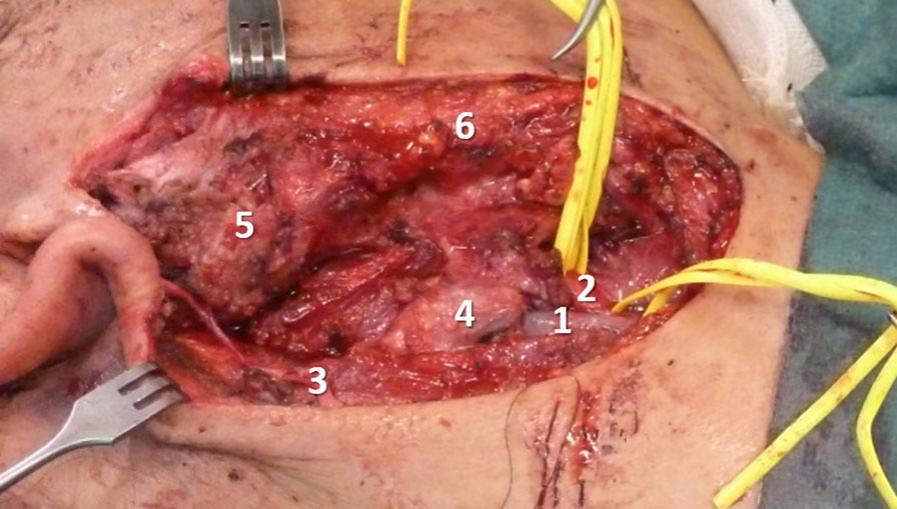
Intraoperative photograph illustrating the exposure of the external carotid artery after its origin in the carotid triangle. *1* Internal jugular vein, *2* External carotid artery, *3* Great auricular nerve, *4* Kuttner lymph node, *5* Superficial lobe of parotid gland, *6* Subcutaneous musculoaponeurotic system of the face

However, in the operating theater, after the compressive dressing was removed, a large arterial bleeding was noted. Immediately, the bleeding was tamponaded, and a preauricular incision was made just deep to the subcutaneous musculoaponeurotic system of the face to expose the parotid gland. This gland presented an opening through which a section of the posterior and lateral flanks of the ECA was identified. To avoid inadvertent injury to the FN, the superficial lobe of the parotid gland was dissected posteriorly to anteriorly, thus exposing the FN and its branches. No lesion of the FN was observed. To minimize arterial bleeding, the incision was extended into a right lateral cervicotomy to expose the ECA after its origin in the carotid triangle (Fig. 2), and this vessel was temporarily clamped. At the same time, the superficial temporal and posterior auricular arteries were digitally compressed at the tragus and at the mastoid apex, respectively, by the surgeon’s assistant. The ECA was repaired deep to the FN with interrupted 6/0 nylon stitches.

**Fig. 3:**
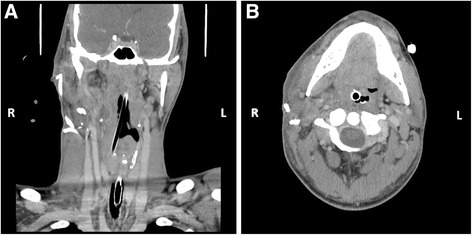
Computed tomographic scans of the neck showing edema and copious liquid in the cervical-facial spaces, exerting a mass effect over the airways with complete obliteration at the soft palate level. **a** Coronal section of the head and neck showing marked swelling of the right parotid and mandibulopharyngeal spaces with left deviation of the airway. **b** Axial section of the head at the level of the oropharynx showing marked edema of the right tonsil and soft palate with compromise of airway patency. *L* Left, *R* Right

The surgery took 7 hours in total. At the beginning of the surgery, the patient’s relevant blood test results were as follows: normal coagulation tests and blood sodium, potassium, and chloride; hemoglobin concentration of 7.2 g/dl (normal range 13.0–17.0 g/dl); hematocrit of 26.7% (normal range 40.0–50.0%); red blood cell count of 2.9 million/mm (normal range 4.4–5.9 million/mm); white blood cell count of 8500/mm (normal range 4.5–11.0 × 10^4^/mm); normal differential leukocyte counts; and normal renal and liver function tests. The patient received 3 red blood cell units during surgery because of intraoperative anemia. No further blood transfusions were needed postoperatively. At the end of surgery, the patient presented with the following significant blood tests results: normal coagulation tests and blood sodium, potassium, and chloride; hemoglobin concentration of 10.5 g/dl (normal range 13.0–17.0 g/dl); normal differential leukocyte counts; hematocrit of 31.1% (normal range 40.0–50.0%); red blood cell count of 3.28 million/mm (normal range 4.4–5.9 million/mm); white blood cell count of 9600/mm (normal range 4.5–11.0 × 10^4^/mm); neutrophilia (8,590/mm [normal range 2.0–8.5 × 10^4^/mm]); and normal renal and liver function tests. In the postoperative period, the patient was transferred to an intensive care unit and was nasotracheally intubated and ventilated. On the second day after surgery, a computed tomographic (CT) scan of the head and neck revealed marked edema and copious liquid in the right cervical-facial spaces. This, in turn, exerted a mass effect over the airways, with almost complete obliteration at the soft palate level (Fig. 3). For this reason, the patient was kept intubated for 8 days. On the third postoperative day, the patient developed a salivary fistula through the preauricular wound that was treated conservatively with the placement of a nasogastric tube, a nil by mouth regimen, and compressive dressings. On the seventh day after surgery, saliva emission through the wound ceased. Only then was the nasogastric tube removed and food intake reinstated. On the eighth postoperative day, a CT scan of the head and neck revealed that the airway swelling had largely subsided, and the patient was extubated. He has remained eupneic on atmospheric air ever since. On the ninth day of hospitalization, the patient was transferred to the plastic surgery department. His wounds healed well. Seven days afterward, he was discharged to home with no deficits in the territory innervated by the FN. The results of all blood tests performed at that time were within normal values. No serological or microbiological tests were done during the treatment of the patient.

Two years after surgery, at his last follow-up visit, the patient had an inconspicuous scar and presented with no motor deficits in the territory of the FN. He claimed to be happy with the functional and aesthetic outcomes.

### Cadaveric dissection

To better understand the anatomical rationale of this uncommon clinical case, we dissected the preauricular region of six cadavers (two females and four males) previously injected at the base of the neck with red- and blue-colored latex solutions in the common carotid artery and internal jugular vein, respectively. Injected cadavers were maintained at 4 °C for at least 24 hours before dissections were performed. The cadavers had no history or evidence of prior surgery in the head and neck region. Cadaver age at the time of death was 76.83 ± 6.21 years (ranging from 67 to 86 years).

**Fig. 4:**
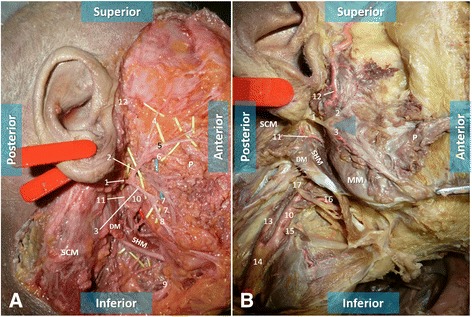
Photographs of anatomical dissections of the right side of the head and neck showing a triangular space (*blue triangle* in **b**) formed by the two main branches of division of the facial nerve. In the bottom of this triangular area, it is possible to observe the anterior flank of the terminal portion the external carotid artery. **a** The skin, the subcutaneous tissue, the superficial musculoaponeurotic system, and the superficial lobe of the parotid gland have been removed to expose the facial nerve and its branches. **b** In addition to the structures removed in (**a**), most of the deep lobe of the parotid gland has been removed to expose the external carotid artery. *SCM* Sternocleidomastoid muscle, *P* Parotid gland, *DM* Digastric muscle, *SHM* Sternohyoid muscle, *MM* Masseter muscle. *1* Facial nerve trunk, *2* Temporal division of the facial nerve (temporofacial trunk), *3* Cervical division of the facial nerve (cervicofacial trunk), *4* Frontal branch of the facial nerve, *5* Zygomatic branches of the facial nerve, *6* Superior buccal branches of the facial nerve, *7* Inferior buccal branches of the facial nerve, *8* Marginal branch of the facial nerve, *9* Cervical branch of the facial nerve, *10* External carotid artery, *11* Posterior auricular artery, *12* Superficial temporal artery, *13* Internal carotid artery, *14* Common carotid artery, *15* Superior thyroid artery, *16* Lingual artery, *17* Hypoglossal nerve

In all cadavers, there was a small triangular space between the two main branches of division of the FN in which the ECA was not covered by the FN. This triangle occurred just before the ECA divided into the maxillary and superficial temporal arteries. The triangle covered the anterior third to the anterior half of this segment of the ECA (Fig. 4a). The angle between the two main branches of the FN (temporofacial and cervicofacial trunks) was measured using ImageJ software (National Institutes of Health, Bethesda, MD, USA). This angle was 98.5 ± 17.5 degrees on the right side and 99.7 ± 17.2 degrees on the left side, being on average 99.1 ± 16.6 degrees (Fig. 4b).

## Discussion

This report describes an uncommon clinical situation in which there was a penetrating injury to the ECA in the parotid region. Reviewing the literature on penetrating injuries of the head and neck with major vascular lesions, we found very few reports of ECA injury in the parotid region, compared with lesions of the carotid and/or vertebral arterial system in the neck [2, 3, 6, 12, 17–21]. Moreover, Tachmes *et al.*, in reviewing parotid gland trauma in a tertiary referral center over a 10-year period, found only four cases of FN injury. In none of these cases was ECA injury present [10]. Furthermore, in the present case report, we describe a very unlikely clinical situation in which the ECA was injured without involvement of the FN. It is well known that when the ECA enters the parotid gland, it lies deep to the FN [22]. Thus, the initial clinical presentation of a large preauricular arterial bleeding with no significant motor deficits in the territory of the FN suggested an arterial lesion superficial to this nerve [22–27].

**Fig. 5:**
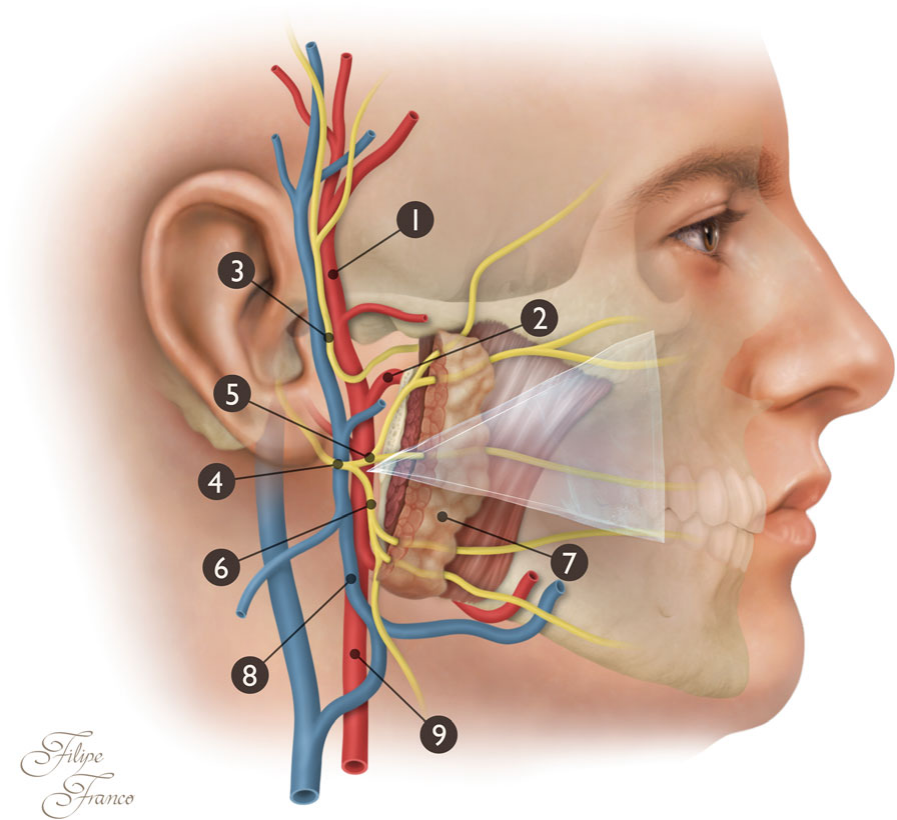
Schematic drawing representing the trajectory of the broken glass in the right preauricular region. The broken glass was able to reach the external carotid artery without sectioning the facial nerve or any other significant neurovascular structures by passing through the triangular space delimited by the temporal and cervical branches of the facial nerve. *1* Superficial temporal artery, *2* Maxillary artery, *3* Auriculotemporal nerve, *4* Facial nerve trunk, *5* Temporal division of the facial nerve (temporofacial trunk), *6* Cervical division of the facial nerve (cervicofacial trunk), *7* Parotid gland, *8* Retromandibular gland, *9* External carotid artery

The cadaveric study that we conducted revealed a small triangular space between the two main branches of division of the FN in which the ECA was not covered by the FN. In the case described, the glass fragment pierced this space and reached the deeper ECA (Fig. 5). This is obviously very improbable. We believe this to be the reason why we found no other such case in the literature, despite our best efforts. As far as we could determine, this triangular space has not been described before [22, 25, 28].

Even today, penetrating injuries to the head and neck region associated with significant arterial bleeding constitute surgical emergencies that are frequently very difficult to handle [2, 3, 5, 29]. The literature suggests that about 25% of penetrating neck trauma cases are associated with vascular injuries, with the internal carotid being the most frequently affected artery [5, 30]. In a 10-year retrospective study in which authors reported the data of 401 patients with penetrating neck trauma, the ECA was the third mostly commonly injured vessel, after the internal carotid and vertebral arteries [31]. Demetriades *et al.*, reporting 223 patients in another large retrospective study, demonstrated that penetrating injuries to the ECA were less common than those involving the internal carotid, common carotid, and vertebral arteries [2, 3].

In contemporary times, penetrating injuries to the head and neck are much more common in war zones and in countries with higher violence rates [3, 4, 6, 12, 14, 18, 20, 21, 30, 32–44]. In other countries, iatrogenic injuries are the main culprits for ECA lesions. Medical procedures most commonly involved in ECA injury are orthognathic surgery, head and neck oncological surgery, and temporomandibular joint invasive procedures [29, 45].

Historically, penetrating injuries to the head and neck were frequently associated with wars. These wounds have been associated with significant mortality rates. For example, during the American Civil War, it is estimated that 15% of the more than 4000 soldiers with these wounds died [1]. Mortality rates dropped to 11% in World War I and to approximately 7% in World War II, then remaining stable around this level for the remainder of the 20th century [6, 8, 12, 18, 20]. Recently, researchers in the Joint Facial and Invasive Neck Trauma (J-FAINT) Project, Iraq and Afghanistan, reported 37,523 facial and penetrating neck injuries in 7177 service members during the period of 2003–2011. These injuries were associated with an overall mortality rate of 3.5%.

It is widely believed that in the observed reduction of mortality, one of the most important contributing factors was the mandatory exploration of all wounds deep to the platysma muscle practiced by most military surgeons since World War II [1]. In 1956, Fogelman and Stewart published a seminal paper in the field, reporting a 6% mortality rate among patients who underwent immediate surgical exploration. This was in stark contrast to the astounding 35% mortality rate among patients who were not treated surgically or whose surgery was initially postponed [46]. Since then, in most trauma centers, mandatory exploration of deep penetrating head and neck injuries has become the gold standard for treatment [1].

Prompt control of bleeding in an ECA lesion is of paramount importance to prevent hemodynamic instability and eventual death [47]. Hemostasis should ideally be achieved through vessel exposure and direct suture of the severed segment [47]. However, this is not always possible, owing to difficulty in surgical access or to the extent of the vascular damage, which may require the use of autologous or synthetic vascular grafts [47]. In cases of difficult access to the severed segment or in cases of multisegment injuries, definitive control of bleeding can be achieved with ECA ligation or selective embolization [8, 47]. The choice is often determined by the availability of a skilled interventional radiologist and the experience of the surgeon in performing ECA ligation [8]. There are many ways to perform an ECA ligature. Most involve ligation of the vessel at its proximal portion just distal to the bifurcation of the common carotid artery. Ligation of the ECA at this level is estimated to reduce blood flow by approximately three-fourths [29]. The concomitant ligation of the superior thyroid, ascending pharyngeal, lingual, and facial arteries has been shown to reduce hemorrhage by roughly 85%. When the posterior auricular artery is additionally ligated, hemorrhage is seen to decrease to approximately 99% of the original carotid blood flow [29]. Selective ECA catheterization and embolization provide the significant advantage of avoiding the morbidity and iatrogenic potential associated with surgical exposure and manipulation of the ECA and surrounding structures. However, they also require catheterization of a large vessel, usually the femoral artery, as well as the use of a contrast agent to detect the bleeding sites and to deposit a thrombogenic agent. All these steps can have potential complications. In addition, selective embolization can be technically challenging in cases of small and tortuous vessels, particularly in the presence of hemorrhage-induced vasospasm [29].

The surgery in the clinical case presented in this report had an unusual level of complexity because the intact FN and its branches represented a fragile nerve mesh over the ECA bleeding site deep in the parotid gland. This made surgical access to the severed artery particularly difficult. Moreover, the numerous anastomoses between the branches of the two ECAs and their neighboring arteries made complete hemorrhage control using proximal arterial clamping impossible [16, 29, 48].

## Conclusions

To the best of our knowledge, this is the first report in the literature of a section of the ECA in the preauricular region without involvement of the FN. The cadaveric dissection study we performed demonstrates that the anatomical basis for this clinical scenario is the existence of a triangular space between the cervicofacial and temporofacial nerve trunks, in which the ECA is not covered by the FN or its branches.

Furthermore, we believe that this case report eloquently demonstrates that in the presence of major penetrating injuries to the head and neck, sound anatomical knowledge is instrumental in establishing a presumptive diagnosis of the severed structures and of the level of the injury. This knowledge is also the basis for instituting adequate therapeutic measures to prevent potentially fatal hemorrhage while preserving functionally significant structures such as the FN [49–56]. This avoided the subsequent need to repair the FN either primarily or with resort to nerve grafts or nerve flaps [24, 57].

## Abbreviations

CT: Computed tomographic
DM: Digastric muscle
ECA: External carotid artery
FN: Facial nerve
J-FAINT: Joint Facial and Invasive Neck Trauma () Project MM, Masseter muscle
P: parotid gland
SCM: Sternocleidomastoid muscle
SHM: Sternohyoid muscle

## References

[1] Atta HM, Walker ML. Penetrating neck trauma: lack of universal reporting guidelines. Am Surg. 1998;64:222–5. 9520810

[2] Demetriades D, Asensio JA, Velmahos G, Thal E. Complex problems in penetrating neck trauma. Surg Clin North Am. 1996;76:661–83. 10.1016/s0039-6109(05)70475-8 8782468

[3] Demetriades D, Theodorou D, Cornwell E, Berne TV, Asensio J, Belzberg H, *et al.* Evaluation of penetrating injuries of the neck: prospective study of 223 patients. World J Surg. 1997;21:41–8. 10.1007/s002689900191 8943176

[4] Kendall JL, Anglin D, Demetriades D. Penetrating neck trauma. Emerg Med Clin North Am. 1998;16:85–105. 10.1016/s0733-8627(05)70350-3 9496316

[5] Irish JC, Hekkenberg R, Gullane PJ, Brown DH, Rotstein LE, Neligan P, *et al.* Penetrating and blunt neck trauma: 10-year review of a Canadian experience. Can J Surg. 1997;40:33–8. PMC3949876 9030081

[6] Brennan JA, Meyers AD, Jafek BW. Penetrating neck trauma: a 5-year review of the literature, 1983 to 1988. Am J Otolaryngol. 1990;11:191–7. 10.1016/0196-0709(90)90037-v 2200295

[7] Ball CG. Penetrating nontorso trauma: the head and the neck. Can J Surg. 2015;58:284–5. 10.1503/cjs.012814 PMC4512872 26022154

[8] Herrera DA, Vargas SA, Dublin AB. Endovascular treatment of penetrating traumatic injuries of the extracranial carotid artery. J Vasc Interv Radiol. 2011;22:28–33. 10.1016/j.jvir.2010.09.022 21109458

[9] Schuenke M, Schulte E, Schumacher U. Topographical anatomy. In: Ross LM, Lamperti ED, Taub E, editors. Atlas of anatomy: head and neuroanatomy, vol. 1. Stuttgart, Germany: Thieme; 2007. p. 92–9.

[10] Tachmes L, Woloszyn T, Marini C, Coons M, Eastlick L, Shaftan G, *et al.* Parotid gland and facial nerve trauma: a retrospective review. J Trauma. 1990;30:1395–8. 10.1097/00005373-199011000-00013 2231807

[11] Kouyoumdjian JA. Peripheral nerve injuries: a retrospective survey of 456 cases. Muscle Nerve. 2006;34:785–8. 10.1002/mus.20624 16881066

[12] Feldt BA, Salinas NL, Rasmussen TE, Brennan J. The Joint Facial and Invasive Neck Trauma (J-FAINT) project, Iraq and Afghanistan 2003-2011. Otolaryngol Head Neck Surg. 2013;148:403–8. 10.1177/0194599812472874 23314163

[13] Madsen AS, Laing GL, Bruce JL, Oosthuizen GV, Clarke DL. An audit of penetrating neck injuries in a South African trauma service. Injury. 2016;47:64–9. 10.1016/j.injury.2015.07.032 26264880

[14] Lundy JB, Cohn SM. Stab wound to the carotid artery. In: Rabinovici R, Frankel HL, Kirton O, editors. Trauma, critical care and surgical emergencies: a case and evidence-based textbook. London: CRC Press/Informa Healthcare; 2010. p. 52–9.

[15] Li L, Li H, Yang K. Multidisciplinary team treatment of penetrating head and neck trauma. J Craniofac Surg. 2016;27:e534–6. 10.1097/SCS.0000000000002850 PMC5023766 27428914

[16] Rodriguez-Luna MR, Guarneros-Zarate JE, Hernandez-Mendez JR, Tueme-Izaguirre J, Noriega-Usi VM, Fenig-Rodriguez J. Defining zone I of penetrating neck trauma: a surgical controversy in the light of clinical anatomy. J Trauma Acute Care Surg. 2016;80:670–3. 10.1097/TA.0000000000000978 26808025

[17] Bodanapally UK, Dreizin D, Sliker CW, Boscak AR, Reddy RP. Vascular injuries to the neck after penetrating trauma: diagnostic performance of 40- and 64-MDCT angiography. AJR Am J Roentgenol. 2015;205:866–72. 10.2214/AJR.14.14161 26397338

[18] Brennan J, Lopez M, Gibbons MD, Hayes D, Faulkner J, Dorlac WC, *et al.* Penetrating neck trauma in Operation Iraqi Freedom. Otolaryngol Head Neck Surg. 2011;144:180–5. 10.1177/0194599810391628 21493412

[19] Khadivi E, Bakhshaee M, Khazaeni K. A rare penetrating neck trauma to zone III. Emerg Med J. 2007;24:840. 10.1136/emj.2006.044586 PMC2658355 18029516

[20] Mahmoodie M, Sanei B, Moazeni-Bistgani M, Namgar M. Penetrating neck trauma: review of 192 cases. Arch Trauma Res. 2012;1:14–8. 10.5812/atr.5308 PMC3955934 24719835

[21] Nason RW, Assuras GN, Gray PR, Lipschitz J, Burns CM. Penetrating neck injuries: analysis of experience from a Canadian trauma centre. Can J Surg. 2001;44:122–6. PMC3695107 11308235

[22] Phillips CD, Bubash LA. The facial nerve: anatomy and common pathology. Semin Ultrasound CT MR. 2002;23:202–17. 10.1016/s0887-2171(02)90047-8 12168997

[23] Adkins WY, Osguthorpe JD. Management of trauma of the facial nerve. Otolaryngol Clin North Am. 1991;24:587–611. 1762778

[24] Barrs DM. Facial nerve trauma: optimal timing for repair. Laryngoscope. 1991;101:835–48. 10.1288/00005537-199108000-00007 1865732

[25] Freilinger G, Gruber H, Happak W, Pechmann U. Surgical anatomy of the mimic muscle system and the facial nerve: importance for reconstructive and aesthetic surgery. Plast Reconstr Surg. 1987;80:686–90. 10.1097/00006534-198711000-00005 3671560

[26] Hanna DC, Gaisford JC. Facial nerve management in tumors and trauma. Plast Reconstr Surg. 1965;35:445–56. 10.1097/00006534-196505000-00001 14288724

[27] May M. Trauma to the facial nerve. Otolaryngol Clin North Am. 1983;16:661–70. 6634187

[28] Lysek Jr MC. Anatomy of the facial nerve. In: Tubbs RS, Rizk E, Shoja MM, Loukas M, Barbaro N, Spinner RJ, editors. Nerves and nerve injuries. Vol. 1: History, embryology, anatomy, imaging, and diagnosis. London: Academic Press/Elsevier; 2015. p. 357–64.

[29] Bouloux GF, Perciaccante VJ. Massive hemorrhage during oral and maxillofacial surgery: ligation of the external carotid artery or embolization? J Oral Maxillofac Surg. 2009;67:1547–51. 10.1016/j.joms.2009.03.014 19531434

[30] Sperry JL, Moore EE, Coimbra R, Croce M, Davis JW, Karmy-Jones R, *et al.* Western Trauma Association critical decisions in trauma: penetrating neck trauma. J Trauma Acute Care Surg. 2013;75:936–40. 10.1097/TA.0b013e31829e20e3 24256663

[31] Sclafani SJ, Cavaliere G, Atweh N, Duncan AO, Scalea T. The role of angiography in penetrating neck trauma. J Trauma. 1991;31:557–63. 10.1097/00005373-199104000-00016 2020041

[32] Cooper A, Barlow B, Niemirska M, Gandhi R. Fifteen years’ experience with penetrating trauma to the head and neck in children. J Pediatr Surg. 1987;22:24–7. 10.1016/s0022-3468(87)80008-8 3819988

[33] Siau RT, Moore A, Ahmed T, Lee MS, Tostevin P. Management of penetrating neck injuries at a London trauma centre. Eur Arch Otorhinolaryngol. 2013;270:2123–8. 10.1007/s00405-012-2324-9 23263269

[34] Bell RB, Osborn T, Dierks EJ, Potter BE, Long WB. Management of penetrating neck injuries: a new paradigm for civilian trauma. J Oral Maxillofac Surg. 2007;65:691–705. 10.1016/j.joms.2006.04.044 17368366

[35] McConnell DB, Trunkey DD. Management of penetrating trauma to the neck. Adv Surg. 1994;27:97–127. 8140981

[36] Ordog GJ. Penetrating neck trauma. J Trauma. 1987;27:543–54. 10.1097/00005373-198705000-00015 3573111

[37] Brett RH, Lu PK, Aw CY. Penetrating neck trauma from nail guns. Singapore Med J. 1998;39:217–9. 9713228

[38] Stone Jr ME, Farber BA, Olorunfemi O, Kalata S, Meltzer JA, Chao E, *et al.* Penetrating neck trauma in children: an uncommon entity described using the National Trauma Data Bank. J Trauma Acute Care Surg. 2016;80:604–9. 10.1097/TA.0000000000000976 26808024

[39] Kim MK, Buckman R, Szeremeta W. Penetrating neck trauma in children: an urban hospital’s experience. Otolaryngol Head Neck Surg. 2000;123:439–43. 10.1067/mhn.2000.109760 11020182

[40] McCrary HC, Nielsen TJ, Goldstein SA. Penetrating neck trauma: an unusual case presentation and review of the literature. Ann Otol Rhinol Laryngol. 2016;125:682–6. 10.1177/0003489416646116 27117904

[41] Lourencao JL, Nahas SC, Margarido NF, Rodrigues Jr AJ, Birolini D. Penetrating trauma of the neck: prospective study of 53 cases [in Portuguese]. Rev Hosp Clin Fac Med Sao Paulo. 1998;53:234–41. 10436632

[42] Apffelstaedt JP, Muller R. Results of mandatory exploration for penetrating neck trauma. World J Surg. 1994;18:917–20. 10.1007/BF00299107 7846919

[43] Merion RM, Harness JK, Ramsburgh SR, Thompson NW. Selective management of penetrating neck trauma: cost implications. Arch Surg. 1981;116:691–6. 10.1001/archsurg.1981.01380170163029 7235962

[44] Burda TM, Cotton BA. Straight for the jugular: managing blunt & penetrating neck trauma in the field. JEMS. 2007;32:40–6. 49. 10.1016/S0197-2510(07)72293-3 17683776

[45] North CM, Ahmadi J, Segall HD, Zee CS. Penetrating vascular injuries of the face and neck: clinical and angiographic correlation. AJR Am J Roentgenol. 1986;147:995–9. 10.2214/ajr.147.5.995 3490169

[46] Fogelman MJ, Stewart RD. Penetrating wounds of the neck. Am J Surg. 1956;91:581–96. 10.1016/0002-9610(56)90289-6 13302565

[47] Rubio PA, Reul Jr GJ, Beall Jr AC, Jordan Jr GL, DeBakey ME. Acute carotid artery injury: 25 years’ experience. J Trauma Acute Care Surg. 1974;14:967–73. 10.1097/00005373-197411000-00010 4419022

[48] Hyde F. Ligation of the external carotid artery for the control of idiopathic nasal hemorrhage. Laryngoscope. 1925;35:899–902.

[49] Atteberry LR, Dennis JW, Menawat SS, Frykberg ER. Physical examination alone is safe and accurate for evaluation of vascular injuries in penetrating zone II neck trauma. J Am Coll Surg. 1994;179:657–62. 7952477

[50] Biffl WL, Moore EE, Rehse DH, Offner PJ, Franciose RJ, Burch JM. Selective management of penetrating neck trauma based on cervical level of injury. Am J Surg. 1997;174:678–82. 10.1016/s0002-9610(97)00195-5 9409596

[51] Burgess CA, Dale OT, Almeyda R, Corbridge RJ. An evidence based review of the assessment and management of penetrating neck trauma. Clin Otolaryngol. 2012;37:44–52. 10.1111/j.1749-4486.2011.02422.x 22152036

[52] Gerst PH, Sharma SK, Sharma PK. Selective management of penetrating neck trauma. Am Surg. 1990;56:553–5. 2393198

[53] Kaya KH, Koc AK, Uzut M, Altintas A, Yegin Y, Sayin I, *et al.* Timely management of penetrating neck trauma: report of three cases. J Emerg Trauma Shock. 2013;6:289–92. 10.4103/0974-2700.120382 PMC3841539 24339665

[54] Kesser BW, Chance E, Kleiner D, Young JS. Contemporary management of penetrating neck trauma. Am Surg. 2009;75:1–10. 19213388

[55] Thompson EC, Porter JM, Fernandez LG. Penetrating neck trauma: an overview of management. J Oral Maxillofac Surg. 2002;60:918–23. 10.1053/joms.2002.33863 12149739

[56] Azuaje RE, Jacobson LE, Glover J, Gomez GA, Rodman Jr GH, Broadie TA, *et al.* Reliability of physical examination as a predictor of vascular injury after penetrating neck trauma. Am Surg. 2003;69:804–7. 14509332

[57] Gosain AK, Matloub HS. Surgical management of the facial nerve in craniofacial trauma and long-standing facial paralysis: cadaver study and clinical presentations. J Craniomaxillofac Trauma. 1999;5:29–37. 11951222

